# E2F1, a Novel Regulator of Metabolism

**DOI:** 10.3389/fendo.2017.00311

**Published:** 2017-11-10

**Authors:** Pierre-Damien Denechaud, Lluis Fajas, Albert Giralt

**Affiliations:** ^1^Center for Integrative Genomics, University of Lausanne, Lausanne, Switzerland

**Keywords:** E2F1, cell cycle regulators, cancer metabolism, obesity, metabolic diseases

## Abstract

In the past years, several lines of evidence have shown that cell cycle regulatory proteins also can modulate metabolic processes. The transcription factor E2F1 is a central player involved in cell cycle progression, DNA-damage response, and apoptosis. Its crucial role in the control of cell fate has been extensively studied and reviewed before; however, here, we focus on the participation of E2F1 in the regulation of metabolism. We summarize recent findings about the cell cycle-independent roles of E2F1 in various tissues that contribute to global metabolic homeostasis and highlight that E2F1 activity is increased during obesity. Finally, coming back to the pivotal role of E2F1 in cancer development, we discuss how E2F1 links cell cycle progression with different metabolic adaptations required for cell growth and survival.

## Introduction: A Cell Cycle Protein with New Skills

The E2F transcription factors were first identified as proteins that were able to bind to the promoter of the adenoviral gene E2 (1). Eight E2F genes (*E2F1-8*) have been described to date, which can be classified based on their protein structures, their interaction partners, and their transcriptional properties (2). E2F1 was the first member of the E2F family to be identified because of its ability to bind the retinoblastoma protein (pRB), a tumor suppressor mutated in many types of cancer (3, 4). The activity of E2F1 is dependent on its binding partners, which include dimerization proteins (DP) and the retinoblastoma family proteins (also known as “pocket proteins”), composed by pRB (*RB1*), p107 (*RBL1*), and p130 (*RBL2*) (5). E2F1–pRB interaction blocks the transcriptional activation domain of the E2F1–DP complex and prevents the recruitment of transcriptional co-activators to the promoters of its target genes (6). During cell cycle progression, cyclin-dependent kinases (CDKs) phosphorylate pRB, releasing E2F1, which is then available to promote the expression of genes involved in S-phase entry, DNA synthesis, and mitosis (7–9).

Three decades after its discovery, it is now clear that the control of cell cycle represents only a subset of the E2F1 roles, which include the regulation of apoptosis (10), senescence (11), and DNA-damage response (12). Indeed, genome-wide location studies have revealed that E2F1 binds to hundreds of promoter regions of genes involved in a myriad of cellular pathways (13–16). What ultimately determines E2F1 distinct biological functions are its protein levels, the combination of several posttranscriptional modifications and its interaction with different partners (17). The intricate role of E2F1 as a master regulator of cell fate has been extensively examined before and is out of scope for this review (17, 18). Instead, here, we want to focus on the recent research evidencing that E2F1 is a master regulator of metabolism both in normal and pathological conditions.

## E2F1 Regulates Metabolism in Non-Proliferative Conditions and Contributes to Global Metabolic Homeostasis

### Role of E2F1 in Normal Physiology

**Figure 1:**
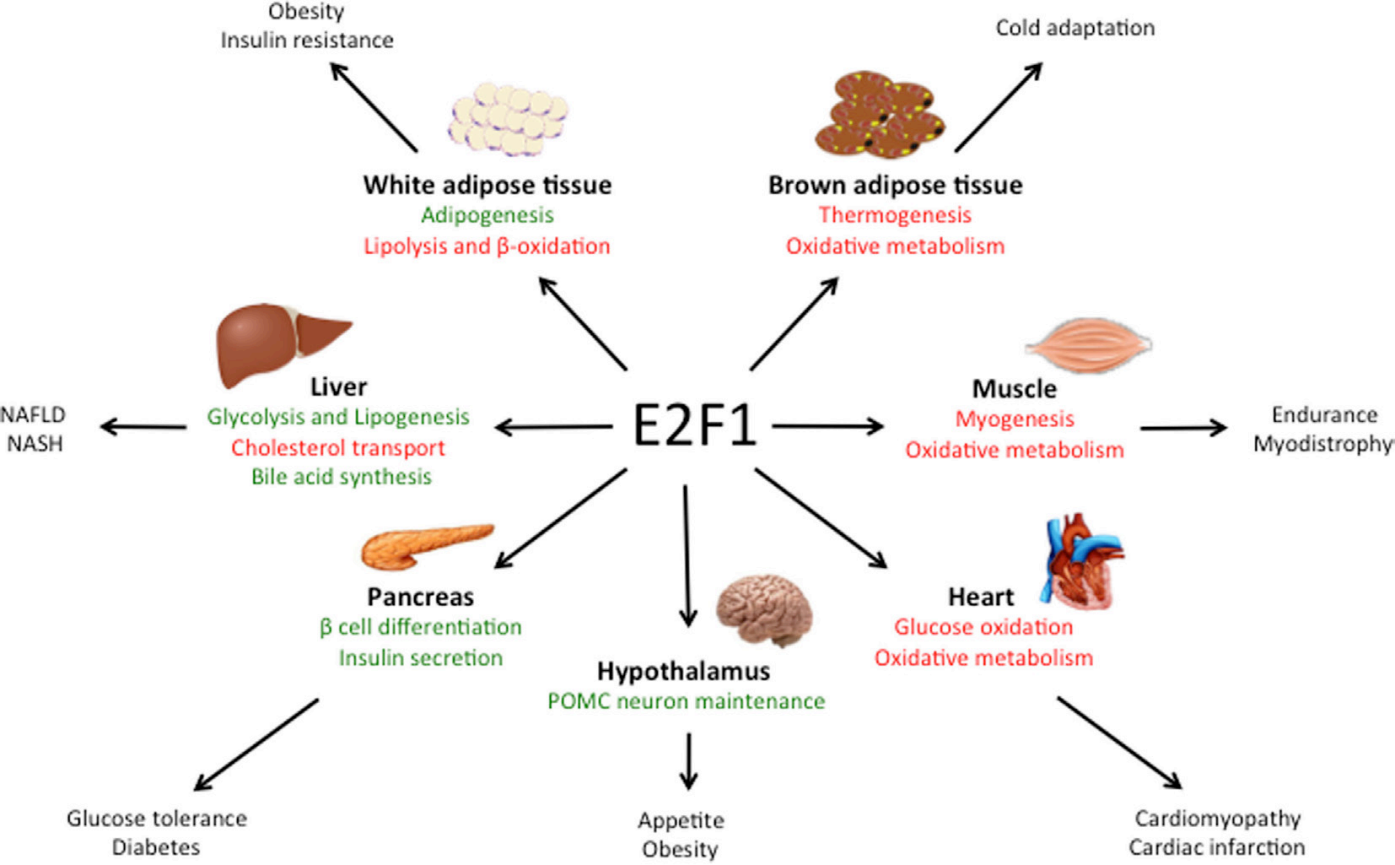
Main roles of E2F1 in metabolic tissues. E2F1 participates in the differentiation of several tissues, but also in the regulation of specific metabolic functions in fully differentiated organs, thus contributing to global metabolic homeostasis. Moreover, during obesity, E2F1 activity is increased and it contributes to some of the comorbidities of this pathological condition. Pathways activated by E2F1 are represented in green while pathways repressed by E2F1 are in red.

Despite the critical function of E2F1 in cell proliferation, *E2f1^−/−^* mice undergo normal development, likely due to the compensation by other E2F family members (19, 20). However, *E2f1^−/−^* mice present some metabolic perturbations that highlight its specific role in the regulation of metabolism independently from cell cycle control. E2F1 participates in the development and the differentiation of several tissues involved in global metabolic homeostasis, but it is also implicated in specific metabolic functions of fully differentiated organs like pancreas, adipose tissues, muscle and liver (Figure 1).

*E2f1/E2f2* mutant mice show severe exocrine atrophy of pancreatic β cells, primarily resulting from E2F1 mutation, which leads to insulin-dependent diabetes (21). E2F1 promotes β cell proliferation and differentiation through the regulation of the endocrine markers PDX-1 and Neurogenin 3 (22, 23). In addition, in fully differentiated β cells, E2F1 directly controls the expression of the major subunit of the ATP-sensitive K^+^ channel Kir6.2, hence promoting glucose-stimulated insulin secretion (24). These studies show that E2F1 participates in pancreas development, maintenance, and endocrine function, hence contributing to global glucose homeostasis.

In the adipose tissue, E2F1 promotes adipogenesis though the regulation of *PPARG* and *RIP140* gene expression, two master regulators of adipocyte fate and differentiation (25, 26). Moreover, in mature adipocytes E2F1 can form a repressor complex with TRIP-Br2—a transcriptional co-regulator—which inhibits lipolysis and mitochondrial β-oxidation (27). Interestingly, CDK4, the main E2F1 upstream activator during cell cycle progression, also promotes adipogenesis though PPARG activation and in mature adipocytes it sustains insulin signaling by phosphorylation of the Insulin Receptor Substrate 2(28, 29). Altogether, these findings show that the canonical CDK4-pRB-E2F1 axis is essential for adipogenesis and to maintain adipocyte function.

In contrast to white adipose tissue, E2F1 represses mouse myogenic differentiation by inhibiting the transcription factors MyoD and Myogenin (30, 31). MyoD in turn, promotes the expression of the Kelch Repeat and BTB Domain Containing Protein 5 (Kbtbd5), which represses E2F1 activity in a negative feedback loop by the ubiquitination and degradation of DP1 (32). Conversely, in *Drosophila*, depletion of the dE2F1 blunts the expression of late myogenic markers during muscle differentiation, which is critical for survival (33). The differences between the two organisms are puzzling and require further exploration, but they may rely on the fact that in *Drosophila* there are only two E2F isoforms, dE2F1 and dE2F2, which work as activators and repressors of transcription, respectively.

Chromatin immunoprecipitation (ChIP) analysis revealed that in basal conditions E2F1 and pRB form a repressor complex in the promoters of several genes involved in oxidative metabolism and mitochondrial biogenesis in muscle, but also in brown adipose tissue (34). In response to exercise or cold exposure, pRB is phosphorylated in muscle and brown adipose tissue, respectively, and mitochondrial and thermogenic genes are expressed (34, 35). As a consequence, deletion of E2F1 in mice results in better resistance to fatigue during exercise and higher body temperature upon cold stimulation due to increased oxidative metabolism (34). Strikingly, E2F1 depletion in a dystrophic mouse model significantly improved muscle performance by increasing muscle oxidative metabolism (36).

Some studies using pRB lack of function models support the role of the E2F1–pRB complex as a negative regulator of oxidative metabolism. For instance, adipose-specific RB1-deficient mice are resistant to high-fat diet (HFD)-induced obesity and display increased mitochondrial activity in white and brown adipose tissues (37). This was reproduced in RB1-haplosufficient mice (38). However, the HFD-resistant phenotype of RB1-deficient mice could also be attributed to the role of pRB in promoting white versus brown fat cell differentiation (35, 39), as evidenced by the increased expression of the thermogenic protein UCP1 in both white and brown adipose tissue depots (37, 38). Additionally, acute loss of pRB or depletion of p170 increased mitochondrial content and activity in muscle cells (40, 41). Conversely, other studies report that pRB may in fact promote mitochondrial biogenesis. Deletion of *RB1* led to impaired mitochondrial function in myocytes (42) and erythrocytes (43). More recently, it was shown that acute pRB loss in adult mice results in a decreased content of oxidative phosphorylation proteins in the lung and in the colon (44), while RB1 depletion blocked muscle differentiation due to an impairment in oxidative metabolism (45). The above confounding studies evidence the relevance of the E2F1-pRB complex in the control of oxidative metabolism in highly metabolic tissues, but they highlight that its specific function may be context dependent. It should also be taken into account that pRB loss of function also leads to multiple E2F1-independent effects (4). Moreover, the fact that E2F1 can activate or repress its target genes often complicates the understanding of the phenotype of E2f1 knockout models.

### Role of E2F1 in Metabolic Diseases

Obesity is associated with increased risk of developing cardiovascular diseases, type 2 diabetes, and cancer (46). As we will discuss in this section, E2F1 expression and activity are increased during obesity in several tissues involved in metabolic homeostasis, suggesting that E2F1 could contribute to some of the comorbidities of this condition.

*E2f1* mRNA and protein levels are increased in the visceral white adipose tissue of obese human subjects and positively correlated with insulin resistance and circulating free-fatty acids (47). E2F1 expression was also increased in the visceral adipose tissue of two widely used mouse models of obesity: mice fed a HFD and leptin-deficient (ob/ob) mice (48). This effect was reversed when HFD-fed mice were treated with resveratrol, which in parallel decreased body weight gain and the levels of pro-inflammatory cytokines levels in white adipose tissue (49). In addition, pRB levels and repressor activity decrease in white adipose tissue during obesity both in rats and in humans (50), which is consistent with increased E2F1 activity. These evidences are supported by ChIP analysis in human white adipose tissue that revealed increased E2F1 binding to the promoters of stress signaling genes during the progression of obesity (51). Interestingly, E2F1 has been shown to enhance NF-κB-mediated inflammatory response (52, 53). However, the contribution of E2F1 to the inflammation of white adipose tissue during insulin resistance remains to be explored.

Obesity is a well-known inducer of cardiac hypertrophy, which often contributes to heart failure (54). Pathological cardiac hypertrophy occurs in parallel with the development of metabolic inflexibility and a re-activation of the cell cycle machinery (55). Similar to the effects observed in the white adipose tissue, HFD increased E2F1 levels and increased RB phosphorylation in mouse heart. This correlated with elevated expression of the E2F1 transcriptional target pyruvate dehydrogenase kinase 4 (PDK4) (56, 57). PDKs inhibit pyruvate dehydrogenase, blocking pyruvate conversion into acetyl-CoA, which results in decreased glucose oxidation. Hence, upregulation of the E2F1–PDK4 axis during obesity may account for the impairment in glucose oxidation that characterizes cardiomyopathy. Moreover, through the regulation of PINK1 translation via miR-421 expression, E2F1 promotes mitochondrial fragmentation in cardiomyocytes, which can lead to myocardial infarction (58). Additionally, E2F1 has been shown to suppress cardiac neovascularization by downregulating VEGF and PIGF expression. Consequently, *E2f1^−/−^* mice present better cardiac function after myocardial infarction than their wild-type littermates (59). Altogether, these studies suggest that increased E2F1 activity occurring during obesity contributes to the development of cardiomyopathy through the re-entry in the cell cycle and the re-wiring of cardiac metabolism.

Some laboratories, including ours, have recently demonstrated the importance of E2F1 in the physiopathological context of non-alcoholic fatty liver disease (NAFLD), which is highly related to the epidemic of obesity. NAFLD is a progressive disease that starts with a benign accumulation of lipids in the liver (hepatic steatosis) that can develop to non-alcoholic steatohepatitis (NASH) which, in its worst prognosis, can lead to liver fibrosis, cirrhosis, and hepatocarcinoma (60). Hepatic E2F1 levels are increased in steatotic liver in mice but also in humans (16). Additionally, NAFLD correlated with the phosphorylation of pRB in the liver in different mouse models of obesity and diabetes (16, 61), altogether consistent with increased E2F1 activity in these conditions. One major contributor to NAFLD is an increase in hepatic *de novo* lipogenesis, a process in which E2F1 plays an important role. Indeed, E2F1 directly activates the expression of key glycolytic and lipogenic genes and E2F1 depletion protects against NAFLD (16). Remarkably, hepatic E2F1 expression is increased in patients with NASH and in different mouse models of liver fibrosis (62, 63). Perturbed bile acid metabolism and/or cholesterol homeostasis are major contributors to NASH. The importance of E2F1 in bile acid synthesis was found in a mouse model of NASH—bile duct ligation and 3, 5- diethoxycarbonyl-1, 4-dihydrocollidine (DCC) feeding—in which bile acid accumulation in the liver contributes to fibrosis. Indeed, knockout of E2F1 in mice reduced bile acid synthesis, which protected from the development of biliary fibrosis under DCC feeding (62). We also recently revealed that E2F1 participates in cholesterol homeostasis by enhancing the expression of PCSK9, a negative regulator of the LDL receptor and cholesterol uptake (63). Importantly, anti PCSK9 antibodies were recently approved for the treatment of cardiovascular diseases due to their capacity to lower LDL cholesterol levels (64). *E2f1^−/−^* mice present decreased circulating levels of cholesterol as a consequence of increased cholesterol uptake by several tissues, including the liver. However, when subjected to a high cholesterol diet, *E2f1^−/−^* mice presented increased liver fibrosis, likely due to the combination of exacerbated cholesterol uptake and a defect in bile acid secretion (63). Taken together, these studies imply that the convenience of targeting E2F1 to treat liver fibrosis could be context dependent and that this approach requires further investigation. Nevertheless, in humans, the increase of E2F1 during NASH was more substantial than the induction of standard fibrosis markers such as α-SMA and α1-collagen, which suggest that E2F1 could be potentially used as a new diagnostic marker for increased risk of developing liver fibrosis and cirrhosis (62).

Long-term HFD also increased E2F1 protein levels and pRB phosphorylation in hypothalamic Arcuate nucleus neurons, which are involved in global energy balance (65). This in turn led to a de-repression of E2F1-target genes involved in cell cycle regulation and apoptosis. Lu et al. found that the E2F1–pRB repressor complex is necessary for POMC neuron maintenance, whereas specific RB1 depletion in these neurons led to hyperphagia, obesity and diabetic syndrome in an E2F1-dependent manner (65). These results indicated that dysregulation of E2F1 at the central level also contributes to the development of the metabolic syndrome during the progression of obesity.

Altogether, recent work has highlighted the importance of the pRB-E2F1 pathway in the pathophysiology of obesity.

## E2F1 Contributes to the Metabolic Reprogramming of Cancer Cells

Cancer cells adapt their metabolism in order to promote growth, proliferation, survival, and metastasis. The specific metabolic profile of a tumor ultimately depends on the tissue of origin, the oncogenic alterations, the tumor stage, and the tumor microenvironment. Metabolic reprogramming is now considered one of the hallmarks of cancer and selectively targeting tumor metabolism has been proposed in the recent years as a therapeutic strategy to treat cancer (66, 67). Remarkably, some oncogenes such as p53 and Myc regulate cancer metabolism (68, 69) and, as we will discuss in this section, so does E2F1 (Table 1).

**Table 1:** E2F1 contributes to the metabolic reprograming of cancer cells.

	E2F1-target genes	Reference
Nucleotide synthesis	DHFR, TK	(85,86)
Lipid synthesis	FAS	(89)
Glycolysis	PFKB, Sirt6, PDK	(71,72,73,75)
Oxidative metabolism	TOP1MT, EVOVL2, NANOG	(76–78)
Autophagy	v-ATPase, ATG1, DRAM1, MAP1LC3	(91,92)

*E2F1 regulates the expression of several genes that have an impact on cancer metabolism*.

*DHFR, dihydrofolate reductase; TK, thymidine kinase; FAS, fatty acid synthase; PFKB, 6-phosphofructo-2-kinase/fructose-2,6-bisphosphatase; PDK, pyruvate dehydrogenase kinase; Sirt6, Sirtuin 6; TOP1MT, mitochondrial topoisomerase I; EVOVL2, ELOVL fatty acid elongase 2; ATG1, autophagy-related gene-1; MAP1LC3, microtubule-associated protein-1 light chain-3; DRAM, damage-regulated autophagy modulator*.

### E2F1 Contributes to the Warburg Effect

**Figure 2:**
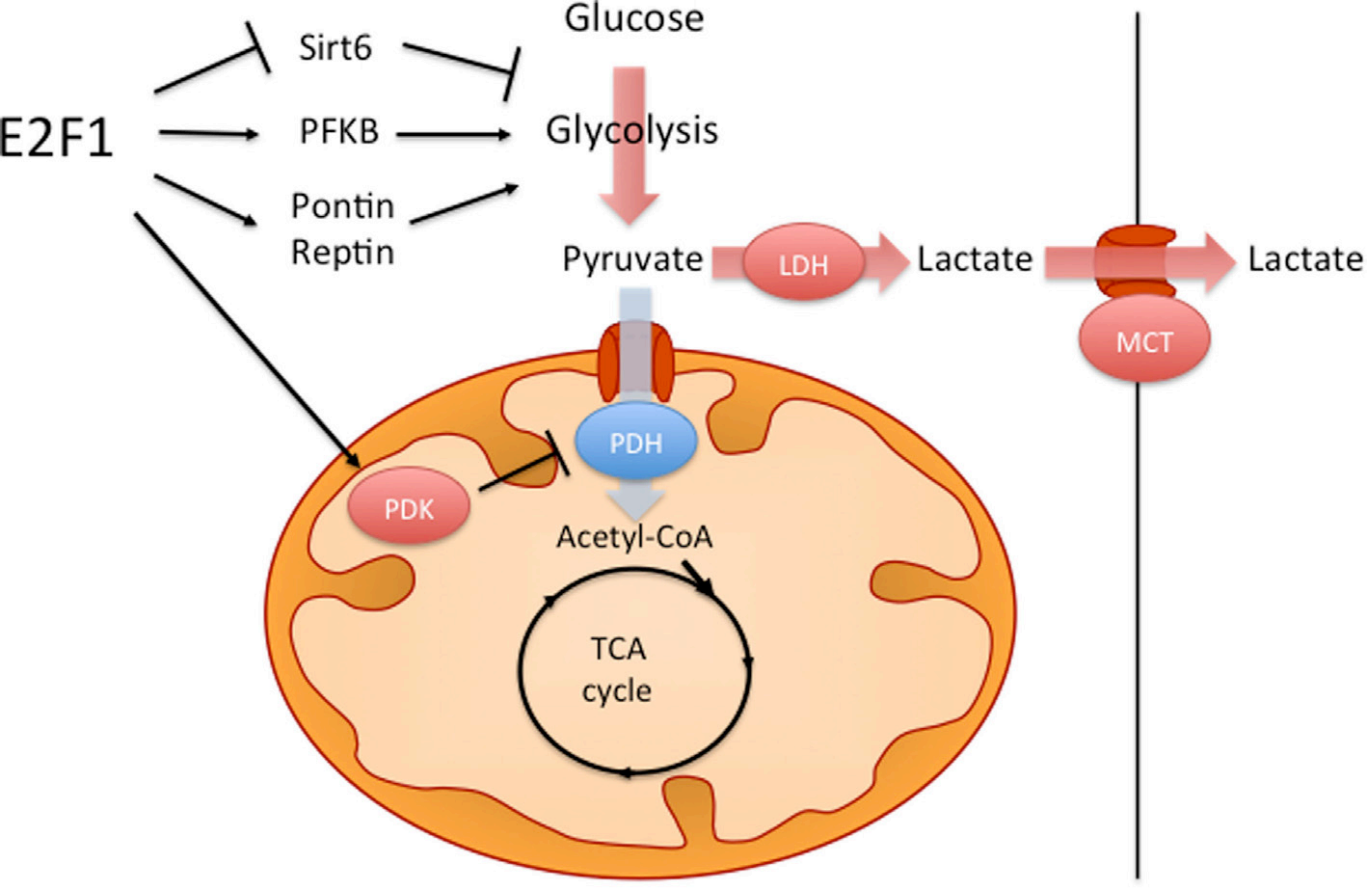
E2F1 contributes to the Warburg effect. E2F1 participates in the characteristic aerobic glycolysis observed in many tumors by different mechanisms. E2F1 promotes glycolysis by repressing the expression of Sirtuin 6 (Sirt6), a negative regulator of glycolytic gene expression and by promoting the expression of the F-type isoform of 6-phosphofructo-2-kinase/fructose-2,6-bissphosphatase (PFKB). E2F1 also recruits a Pontin/Reptin complex to promote the expression of genes involved in glycolysis and lactate export. Additionally, E2F1 blocks glucose oxidation in the mitochondria by promoting the expression of pyruvate dehydrogenase kinase (PDK) enzymes, which inhibit the pyruvate dehydrogenase complex (PDH).

One metabolic feature of many cancer cells is the so-called Warburg effect, which consists on increased aerobic glycolysis and decreased glucose oxidation, resulting in high rates of glucose utilization and lactate production (66, 70). It has been shown that, against the assumption of Otto Warburg, who first described this phenomenon almost a century ago, in most cancers this is not caused by defective mitochondria. Several hypotheses have been proposed on how the Warburg effect benefits cancer cells, including higher rates of ATP synthesis, the generation of glycolytic intermediates for biosynthetic reactions or the remodeling of the tumor microenvironment; however, this phenomenon is still not fully understood (70). It has been shown that E2F1 can promote this metabolic switch by both enhancing glycolysis and by repressing glucose oxidation in the mitochondria (Figure 2). During the development of HCC, increased E2F1 levels progressively recruit Pontin and Reptin (two putative DNA helicases) to promote the expression of genes involved in glycolysis and in lactate export, which contributes to the Warburg effect (15). During cell division, E2F1 also promotes the expression of the F-type isoform of the enzyme 6-phosphofructo-2-kinase/fructose-2,6-bisphosphatase, which results in the synthesis of fructose-2,6-bisphosphate, a potent stimulator of glycolysis (71, 72). Moreover, E2F1 also enhances glycolysis in bladder and prostate cancer cell lines through the suppression of the expression of Sirtuin 6, a NAD(+)-dependent deacetylase that inhibits the transcription of several key glycolytic genes (73, 74). Besides enhancing glycolytic gene expression, as previously mentioned, E2F1 also blocks glucose oxidation in the mitochondria by promoting the expression of the PDK enzymes. While in the heart E2F1 regulates PDK4 (57), in pancreatic cancer cells E2F1 enhances the expression of PDK1 and PDK3 isoforms, which results in increased aerobic glycolysis and proliferation (75).

### E2F1 and Oxidative Metabolism

In addition to regulating oxidative metabolism in non-proliferative conditions (34), E2F1 also repress mitochondrial biogenesis during proliferation. Like in the muscle, knocking down E2F1 in HeLa cells led to increased expression of several genes involved in mitochondrial biogenesis and oxidative phosphorylation, which resulted in increased ATP production (76). E2F1 depletion in Mesenchymal Stem Cells also increased mitochondrial biogenesis and oxygen consumption (77). Additionally, it has been shown that E2F1-mediated repression of oxidative metabolism results in a self-renewal of tumor-initiating stem-like cells that contributes to the progression of HCC (78). Some evidences show that mitochondrial function, in turn, also impacts E2F1 activity. For instance, inhibition of ATP synthase or of the electron transport chain leads to the downregulation of E2F1 activity and to cell cycle arrest (79, 80). On the other hand, mitochondrial ROS production can promote E2F1-mediated apoptosis (81, 82). For a more detailed perspective of the complex interplay between E2F transcription factors and the mitochondrial function, we address you to recent specific reviews about the topic (83, 84).

### E2F1 and Anabolic Metabolism

Cancer cells undergo different anabolic processes to fulfill the high demand of macromolecules required for proliferation. E2F1 participates in DNA synthesis by regulating the expression of several genes involved in nucleotide metabolism such as Thymidine kinase and Dihydrofolate reductase (85, 86). Tumors also normally present high rates of lipid synthesis, which are used both for membrane production and as signaling molecules (87). Lipogenesis is not only important during proliferation; it also contributes to the metastatic capacity of cancer cells (88). Besides promoting lipogenesis in the liver (16), in medulloblastoma E2F1 enhances fatty acid synthase expression in response to Sonic hedgehog signaling (89).

mTORC1 is a master regulator of cell growth and survival, and it is involved in the progression of many cancers (90). It was recently shown that E2F1 promotes mTORC1 activity by enhancing the expression of lysosomal v-ATPase. This in turn, blocked autophagy, one of the main metabolic processes regulated by mTORC1 (91). Conversely, it was shown that E2F1 can also stimulate upregulation of genes involved in autophagy in response to DNA damage (92). Hence, the contribution of E2F1 to autophagy is still a matter of debate. Additionally, numerous studies have highlighted the crosstalk between E2F1 activity and other signaling pathways involved in cancer metabolism, such as the AKT or the HIF pathways (93–95). Whether E2F1 promotes anabolic reprogramming in cancer cells through the interaction with these signaling pathways remain to be explored.

Overall, these studies show that the transcription factor E2F1 plays a pivotal role integrating the cell cycle regulatory machinery with metabolic pathways essential for cell growth and survival. This, in turn, determines cell fate by affecting cell stemness, proliferation rate, or apoptosis. Therefore, inhibiting E2F1 activity could potentially impact tumor development at different levels simultaneously by blocking cell cycle progression and by impairing metabolic flexibility in cancer cells. In this regard, CDK4/6 inhibitors that block pRB phosphorylation and that are currently used for treating hormone-positive breast tumors have been reported to block proliferation, in part, by inducing a metabolic reprogramming in cancer cells (96, 97).

## Conclusion and Perspectives

Here, we have collected the current and emerging evidence showing that E2F1 regulates metabolism in non-proliferating conditions and, more importantly, that dysregulation of E2F1 activity leads to complications associated with obesity. Many studies have focused on the mitogenic signals that drive E2F1 activation in cancer cells, but how E2F1 is activated in other pathological conditions such as obesity is just beginning to be understood. The CDK4-pRB-E2F1 pathway can be stimulated both by glucose and by insulin in different tissues involved in global metabolic homeostasis (16, 24, 29, 95, 98). One possibility is that during obesity, hyperglycemia and/or hyperinsulinemia render pRB hyperphosphorylated (50, 61, 65). This in turn, would increase E2F1 activity and, in a positive feedback loop, E2F1 could promote its own expression (99). Other possible candidates for exacerbated E2F1 activation during obesity could be chronic inflammation or increased ROS production due to mitochondrial stress, two factors that promote E2F1 activity in other contexts (52, 82). Despite the specific mechanisms that lead to E2F1 hyperactivation during obesity, targeting E2F1 could potentially be used to ameliorate some of the deleterious effects of this condition. Notably, *E2f1^−^*^/^*^−^* mice present increased insulin sensitivity and are resistant to HFD-induced obesity (25, 34). However, it should be considered that systemically inhibiting E2F1 activity would likely impair insulin secretion (100), which could be detrimental in the initial phases of insulin resistance, when insulin production is enhanced to maintain normoglycemia.

Given its dual role in proliferation and metabolism, it is tempting to speculate that E2F1 might be a central actor in the interplay between obesity and some types of cancer. One of those cases could be HCC, for which there is an increased risk in obese patients (101). We have recently shown that hepatic E2F1 expression is augmented during obesity (16), while numerous studies have demonstrated that increased E2F1 activity promotes the development of HCC (15, 78, 102, 103). Notably, it was also recently reported that E2F1 mediates the proliferative effects of insulin in hepatocytes (95). Indeed, obesity-associated hyperinsulinemia is one mechanism proposed to explain the epidemiological observations of increased HCC in obese patients (104). Therefore, under obesity conditions, enhanced hepatic E2F1 activity—maybe in response to hyperinsulinemia—may first lead to enhanced *de novo* lipogenesis, NAFLD development and fibrosis (16, 62). Subsequently, E2F1 may contribute to HCC progression by promoting the expression of genes involved in cell cycle machinery and cancer metabolism (15).

In conclusion, research over the past 15 years has given an increasingly complex picture of the multiple roles of E2F1. Beyond being a mere cell cycle regulator, this transcription factor has emerged as a novel player in the control of metabolism not only in normal physiology but also under pathological conditions such as obesity and cancer.

## Author Contributions

PDD, LF, and AG conceived and wrote the manuscript.

## Conflict of Interest Statement

The authors declare that the research was conducted in the absence of any commercial or financial relationships that could be construed as a potential conflict of interest.
